# Is Frailty a Good Predictor of Postoperative Complications in Elective Abdominal Surgery?—A Single-Center, Prospective, Observational Study

**DOI:** 10.3390/jpm13050869

**Published:** 2023-05-21

**Authors:** Szymon Czajka, Maria Taborek, Łukasz J. Krzych

**Affiliations:** 1 Department of Anaesthesiology and Intensive Care, Faculty of Medical Sciences in Katowice, Medical University of Silesia, 40-752 Katowice, Poland; 2 Students’ Scientific Society, Department of Anaesthesiology and Intensive Care, Faculty of Medical Sciences in Katowice, Medical University of Silesia, 40-752 Katowice, Poland

**Keywords:** frailty syndrome, abdominal surgery, postoperative complications, geriatric anaesthesia

## Abstract

Background: Despite the common occurrence of postoperative complications in patients with frailty syndrome, the nature and severity of this relationship remains unclear. We aimed to assess the association of frailty with possible postoperative complications after elective, abdominal surgery in participants of a single-centre prospective study in relation to other risk classification methods. Methods: Frailty was assessed preoperatively using the Edmonton Frail Scale (EFS), Modified Frailty Index (mFI) and Clinical Frailty Scale (CFS). Perioperative risk was assessed using the American Society of Anesthesiology Physical Status (ASA PS), Operative Severity Score (OSS) and Surgical Mortality Probability Model (S-MPM). Results: The frailty scores failed to predict in-hospital complications. The values of AUCs for in-hospital complications ranged between 0.5 and 0.6 and were statistically nonsignificant. The perioperative risk measuring system performance in ROC analysis was satisfactory with AUC ranging from 0.63 for OSS to 0.65 for S-MPM (*p* < 0.05 for each). Conclusions: The analysed frailty rating scales proved to be poor predictors of postoperative complications in the studied population. Scales assessing perioperative risk performed better. Further studies are needed to obtain optimal predictive tools in senior patients undergoing surgery.

## 1. Introduction

According to data from World Population Prospects, the percentage of people over the age of 65 in the world population will increase significantly from 10% in 2022 to approximately 16% in 2050, by which time 25% of the population of Europe and the United States is likely to be over 65 [1]. Obviously, the average age of patients undergoing surgery will also increase dynamically. The number of people aged 75 years or more undergoing surgery in the United Kingdom increased from 544,998 (14.9% of that age group) in 1999 to 1,012,517 (22.9%) in 2015 [2]. The management of these patients is a challenge for clinicians who are primarily involved in perioperative medicine, i.e., both surgical teams and anaesthetists. Interestingly, there are several different ways of defining old age, and the cut-off point for old age has not been unanimously established. In fact, the United Nations (UN) has defined older people as those aged 60 or over in World Population Ageing 2020 [3], while the World Health Organisation (WHO) has stated that older people in the developed world are commonly defined as people 65 years of age or older. The WHO also uses an alternative definition, in which an older person is someone who has exceeded life expectancy at birth. However, regardless of the cut-off point expressed by numbers, the older age group is remarkably heterogeneous in terms of comorbidity and physical performance [4]. This is where frailty comes into play; although obviously age-related, it involves reduced resilience, loss of adaptability and increased susceptibility to stressors, which may be independent of age. Numerous tools have been developed to assess frailty. Some are based on medical history, others require an examination of the patient and some combine both methods of data collection. Several studies have shown that frailty is a risk factor for a poor prognosis in patients undergoing some types of surgery [5,6]. On the other hand, there is a body of evidence questioning the significance of this method of evaluating patients in this group [7,8,9,10]. Therefore, the ESC/ESA guidelines for non-cardiac surgery recommend the assessment of frailty in patients at risk of it but mainly highlight the prediction of mortality, emphasising that frailty parameters are included in the NSQIP questionnaire [11].

The aim of our study was to assess the association of frailty with possible postoperative complications after abdominal surgery in participants of a single-centre prospective study in relation to other risk classification methods.

## 2. Materials and Methods

### 2.1. Study Design and Patients

We performed a prospective, single-centre, observational cohort study focused on elderly patients admitted to the Gastrointestinal Surgery Department of the University Clinical Centre in Katowice from November 2021 to May 2022. Patients over 60 years of age undergoing elective procedures were eligible for the study. Patients being treated for Parkinson’s disease and taking antidepressants were excluded, as this may interfere with the assessment of frailty [12]. Investigators previously trained in questionnaire administration assessed patients using frailty rating scales according to a predesigned protocol. Data collected during the preoperative anaesthetic consultation were also analysed. Only the subjects admitted for elective procedures were screened. The patients who underwent surgery more than once during the study (even during separate hospital stays) were evaluated before the first procedure. Written informed consent was obtained from the participants. The study was approved by the Bioethics Committee of the Medical University of Silesia in Katowice (no. OCN/CBN/0052/KB/116/22) and no consent to conduct research was necessary due to its noninterventional character (Sections 21 and 22 of the Act of 5 December 1996 on the Medical Profession in Poland). The study was registered in the Research Registry database (UIN researchregistry8709).

### 2.2. Frailty Measures

Three tools using different assessment methods were applied to evaluate frailty. The Edmonton Frail Scale (EFS)—a performance-based multidimensional frailty assessment tool [13], the 11- factors Modified Frailty Index (mFI-11, which is a deficit accumulation model [14], and the Clinical Frailty Scale (CFS)—a clinical judgment-based tool [15]. The EFS measures the following domains: cognition, general health status, functional independence, social support, medication use, nutrition, mood, incontinence and functional performance. Regarding the EFS score, the following categories were distinguished: “not frail” (0–5), “vulnerable” (6–7), “mild frailty” (8–9), “moderate frailty” (10–11) and “severe frailty” (12–17). The mFI-11 provides information on physiological deficits and comorbidities, including clinical conditions such as diabetes mellitus, chronic or acute respiratory failure, congestive heart failure, hypertension that requires pharmacotherapy, peripheral vascular disease, history of angina pectoris, myocardial infarction, stroke or transient ischaemic attack, sensory disorder and functional dependency. To determine the index value, the subject’s score is divided by the total of 11 ranking factors. In our study, frailty was defined as mFI ≥ 0.27. According to the CFS, frailty and fit assessment is based on clinical judgment. The scale classifies patients into one of nine categories, ranging from “very fit” to “terminally ill” in terms of general impression of healthcare providers. A patient was classified as “frail” if the fourth or higher category was achieved. The researchers were trained in CFS application prior to the onset of the study.

### 2.3. Perioperative Risk Measures

Anaesthesia-related patient risk was classified with the standard use of ASA-PS classification. Surgical risk assessment was performed based on the 2022 ESC/ESA guidelines [11]. Surgical risk was also alternatively assessed using the Operative Stress Score (OSS), which assigns a 5-point scale based on procedure-induced physiologic stress, allowing evaluation of outcomes across a broad spectrum of procedures [16]. Global procedural risk was assessed using the Surgical Mortality Probability Model (S-MPM) [17], which was developed for non-cardiac patients and includes the patient risk (according to the ASA-PS classification), procedural risk (according to the ESA/ESC guidelines) and urgency of the procedure (emergency or non-emergency). The S-MPM predicts the risk of early death in three classes: Class I—expected mortality < 0.5%, Class II—expected mortality 1.5–4%, and Class III—expected mortality > 10%.

### 2.4. Follow-Up and Outcome Measures

During post-discharge analysis of subjects’ medical records, the demographic data, comorbidities, adverse events, need for admission to the ICU and length of hospitalisation were collected. According to the ESC guidelines, the surgical risk was defined based on the type of intervention as low-risk procedures (<1%), moderate-risk procedures (1–5%) and high-risk procedures (>5%). The five-grade Clavien–Dindo Classification [18] was applied to rate postoperative complications. The primary outcome of our study was to investigate the relationship between preoperative frailty and the prevalence of complications in the postoperative period among patients undergoing elective gastrointestinal surgery.

### 2.5. Statistical Analysis

Statistical analysis was performed using MedCalc Statistical Software version 18.1 (MedCalc Software Ltd., Ostend, Belgium). Continuous variables were expressed as median and interquartile range (IQR). Qualitative variables were expressed as absolute values and/or percentages. Inter-group differences for quantitative variables were assessed using the Mann–Whitney U-test or Kruskal–Wallis test. Their distribution was verified with the Shapiro–Wilk test. The chi-square test or Fisher’s exact test were applied for qualitative variables. Odds ratios (OR) with their 95% confidence intervals (CI) were calculated, if applicable. All tests were two-tailed. A stepwise logistic regression method was used to verify the findings from bivariate analyses. The occurrence of postoperative complications (according to the Clavien–Dindo classification) was considered the dependent variable. Independent variables were included in the equation if the *p*-value was <0.1 in bivariate comparisons. Appropriate logistic ORs (95% CI) were calculated. The areas under the receiver operating characteristic curves (AUC ROC) were assessed and exact binominal 95% confidence intervals (CI) for the AUCs were calculated. Diagnostic accuracy was defined as unsatisfactory if an AUC was <0.6, satisfactory if an AUC was 0.6–0.69, good if an AUC was 0.7–0.79 and very good if an AUC was at least 0.8. A *p* value of <0.05 was considered statistically significant.

## 3. Results

**Figure 1:**
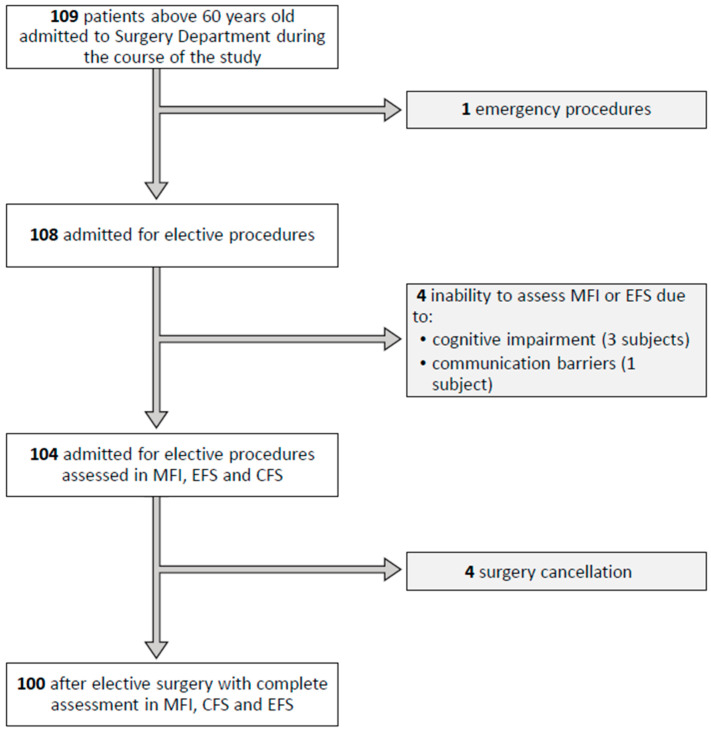
Study flow diagram.

**Figure 2:**
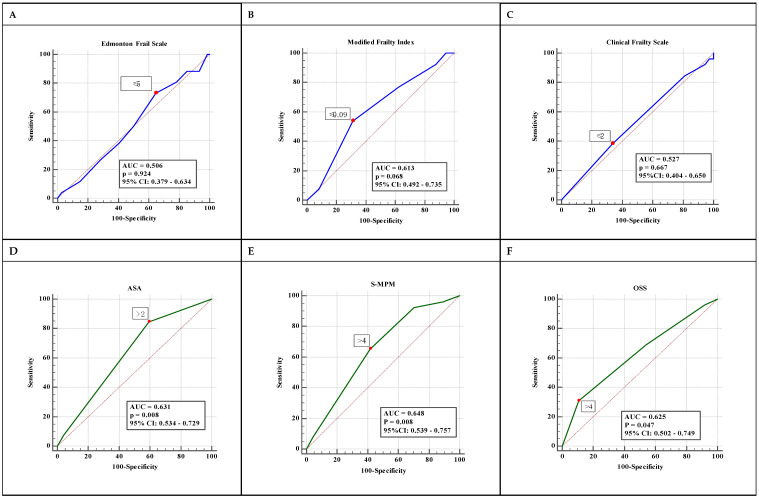
Predicting in-hospital complications and mortality using EFS (**A**), mFI (**B**), CFS (**C**), ASA (**D**), S-MPM (**E**) and OSS (**F**).

One hundred and nine patients at the age of 60 years and older were recruited to the study on the day of hospital admission. Nine subjects were excluded due to communication barriers that prevented interviewing, cancellation of surgery or when the procedure was classified as emergency (Figure 1). Finally, 100 patients were included and analysed. The mean age of the study population was 70 years and the mean BMI was 26.3 kg/m^2^; 57 per cent of participants were female and 66 per cent had an ASA-PS class of three or higher. Twenty-six patients developed a complication assessed as Clavien–Dindo grade ≥ II and five died during hospitalisation. Table 1 presents complete characteristics of the study group. Table 2 summarises the medical and surgical complications that occurred during the study and Table 3 presents the complications in regard to the Clavien–Dindo classification. Considering the Edmonton Frail Scale (EFS), 14 subjects were classified as “frail” and 19 subjects were classified as vulnerable and prone to deterioration. Assessment of the Clinical Frailty Scale (CFS) demonstrated similar results, identifying 18 frail patients. Median CFS score was 3 (2–3). The Modified Frailty Index (mFI) classified 34 patients into the severe frailty group with mFI ≥ 0.27. The median mFI value was 0.18 (0.09–0.27). Frailty measurement instruments did not predict the occurrence of in-hospital postoperative complications, regardless of the character of complication or the Clavien–Dindo grade (Table 4). Table 5 summarises the correlations between the results of the frailty assessment scales tested and the scores from the risk assessment tools. The relationships between the parameters studied and the incidence of complications adre shown in greater detail in Table 6 and Table 7. The risk of surgical procedure contributed to the prevalence of postoperative adverse events. An ASA-PS Class of >2 (AUC ROC 0.631; 95%CI 0.534–0.729, *p* < 0.008), S-MPM score > 4 (AUC ROC 0.648; 95%CI 0.539–0.757, *p* < 0.008) and evaluation in OOS > 4 (AUC ROC 0.625; 95%CI 0.502–0.749, *p* < 0.047) were associated with an increased incidence of complications after surgery. The ASA-PS predicted medical complications (OR 4.67; 95%CI 1.45–15.16, *p* < 0.009). The OOS predicted surgical complications (OR 1.85; 95%CI 1.04–3.63, *p* < 0.047) and the S-MPM predicted both medical and surgical adverse events (OR 2.13; 95%CI 1.08–4.19, *p* < 0.0029 and OR 1.79; 95%CI 1.06–3.29, *p* < 0.042, respectively). The frailty scores failed to predict in-hospital complications (Figure 2A–C). The values of AUCs for in-hospital complications ranged between 0.5 and 0.6 and were statistically nonsignificant. The perioperative risk measuring system performance in ROC analysis was satisfactory (Figure 2D–F), with AUC ranging from 0.63 for OSS to 0.65 for S-MPM (*p* < 0.05 for each). We did not observe any improvement in the predictive values of the scales after adding values derived from the frailty assessment (AUC ROC 0.529–0.587; *p* > 0.05 for each analysis).

## 4. Discussion

### 4.1. Application of Frailty Assessment Scales in a Surgical Patient Population

In this analysis, frailty measurement instruments (The Edmonton Frail Scale (EFS), Modified Frailty Index (MFI) and Clinical Frailty Scale (CFS)) did not predict the occurrence of in-hospital postoperative complications, regardless of the character of complication. The poor predictive value of these scales for in-hospital complications applied to complications in general, as well as medical and surgical complications. In our study, the ASA, S-MPM and OSS proved to be the parameters that allow better estimation of the risk of perioperative complications in gastrointestinal surgery.

The senior population remains ever more firmly in the spotlight of perioperative medicine. While Comprehensive Geriatric Assessment is still the gold standard in frailty assessment [19], its complexity and time-consuming nature limit its use in anaesthesiology or surgery. Therefore, we have chosen three more accessible and implementable frailty measuring systems, which represent different approaches to assessing this phenomenon, i.e., the mFI—a deficit accumulation model, the EFS that also includes a clinician’s assessment of patient performance and the clinical judgment-based CFS. Noteworthy, despite the good correlation of the results of the scales evaluated, all of them did not accurately assess the risk of complications in the surgical population.

The studies published so far differ in terms of the characteristics of the populations studied and, above all, in terms of the treatment spectrum. There are very few articles which evaluate the entirety of elective abdominal interventions in the elderly population; more frequently, research concerns only oncological patients.

### 4.2. In Search for the Optimal Frailty Threshold—Is Frail Really Vulnerable?

The prevalence of frailty in our cohort ranges from 14% to 34%, with mFI demonstrating the highest incidence of frailty syndrome. This finding is consistent with other studies. The meta-analysis including 2281 individuals has estimated the occurrence of frailty in general surgery patients at 10.4% to 37% [20]. According to another meta-analysis focused on oncological, colorectal procedures, the percentage of frail patients ranges from 12 to 56% [9].

Although the 11-item Modified Frailty Index is commonly used for frailty assessment, the lack of consensus on optimal cut-off is noteworthy as a potentially disturbing factor affecting the comparability of results. For instance, Garland et al. [21] have postulated an mFI cut-off of 0.18 in elderly individuals diagnosed with gastrointestinal cancer. According to their findings, this mFI value is associated with increased morbidity; however, it required adjustment for age, sex, ASA category, albumin serum concentration and body mass index, which may preclude assessment in clinical settings. A diametrically different cut-off point for the mFI scale (0.36) was adopted by Sonny et al. [22]. Despite the use of such a high threshold, this study did not demonstrate an association between the presence or absence of frailty and postoperative complications. Another interesting review study on the CFS scale reported (as many as) four different cut-off points used when categorising this variable; a CFS score of five was the most widely used frailty cut point (68.9%) [23]. In this context, it is worth noting that we deliberately did not focus our study on arbitrary frailty thresholds. When a cut-off point is used, the strength of a predictive scale can be assessed by measures of sensitivity and specificity. However, in many cases, the scales are designed to be measured over a continuous or ordinal range. According to Chao et al., the scales tested as continuous variables better predict the mortality in the age group over 50, while dichotomisation of variables results in a risk of bias and loss of many variables that remain on the spectrum between categories [24]. In 2013, Romero-Ortuno proposed matching the Frailty Index cut-off points with the patient’s age, proving that this scale performs differently in different age groups [25]. The recent study by Fronczek et al. in the population of ICU patients concludes that, despite great attractiveness of dichotomising the state of fitness and frailty, it should be remembered that frailty is a continuum [26]. Considering the above, it is desirable to assess the predictive value of the scale over a range of possible cut-off points. Therefore, in our study, ROC curve analysis was used.

### 4.3. Frailty and the Risk of a Negative Outcome after Surgery

Although the association between frailty and the incidence of perioperative complications in some clinical settings appears to be generally well documented, it has been both confirmed [5,6,14,27,28] and questioned [8,9,10,21,22,29] in several studies, as well as meta-analyses. In our study, both univariate logistic regression analysis and ROC curve analysis showed no relationship between postoperative complications and frailty rating scale values. Similar conclusions were drawn from a study by MacLaine et al. [8], where, despite the high prevalence of frailty in the general population of adults undergoing elective surgery, frailty assessment tools showed high sensitivity but low specificity and failed in assessing complications. It should be emphasised that frailty assessment tools were developed to evaluate exposure in the general population, e.g., nursing home patients, and their use in the surgical population should be considered a secondary application. In an interesting study published by Sonny et al., two frailty assessment scales (mFI and the Hopkins Frailty Score) were unable to predict the risk of complications, readmissions or the length of hospital stay. Studied scales did, however, predict the length of hospitalisation only after considering confounding factors in the calculations [22].

In our research, for the reasons mentioned above, we tried to focus on studies applying analysis of the area under the ROC curve. Garland et al. [21] have showed poor performance of the mFI scale in predicting major complications—AUC = 0.5625. Similarly, in a study by Amrock et al., the authors’ frailty assessment scales based on a cohort of NSQIP database (data) scored poorly in the ROC curve analysis in terms of predicting postoperative complications, while mortality was predicted more effectively [30]. In the previously mentioned study by Sonny et al., in the context of ROC curve analysis, both evaluated scales predicted complications poorly and comparably to our data—the AUC ROC was 0.59 for the Modified Frailty Index and 0.60 for the Hopkins Frailty Score [22]. As in our study, in a paper published by Kapoor et al. in 2017, the C-statistic results for predicting perioperative complications were worse for the Frailty Phenotype assessment scale (Frailty Phenotype) than for clinical data (American College of Surgeons Surgical Risk Calculator). Both tools achieved AUROC results similar to those assessed in our study [31]. It is worth mentioning that a large number of studies report good prediction of complications by frailty assessment scales in the case of emergency and orthopaedic surgery [32,33,34]. It can be assumed that this is due to the patient selection. Patients who, due to multi-morbidity and, consequently, also frailty, would not be qualified for elective surgery are often qualified for urgent surgery.

### 4.4. “Traditional” Ways of Risk Assessment

While comparing the applicability of conventional risk assessment methods, such as the ASA class and frailty assessment scales, the characteristics of the study population and the selection of appropriate tools and outcome measures seem to be important. In a study by Kim et al. published in 2014, the multidimensional frailty score based on comprehensive geriatric assessment (CGA) was more useful than ASA PS in predicting outcomes (postoperative mortality and length of hospitalisation) in geriatric patients undergoing surgery (AU ROC 0.821 vs. 0.647; *p* = 0.01) [35]. According to another study by Serrett et al., both mFI and ASA were not associated with complication rates for oncologic and nononcologic urologic surgery, although this study used mFI rather than CGA to assess frailty [36]. In still another study, Yin et al. found out that ASA PS classification was associated with the risk of negative outcome (AU ROC = 0.768) in a population of elderly patients undergoing abdominal surgery; of the scales studied, only the CFS significantly improved the predictive value of ASA classification (AU ROC = 0.815; *p* = 0.048). Despite the relatively good correlation between the ASA PS and frailty assessment scales in our study, no improvement in the accuracy of predicting complications according to the ASA scale was demonstrated when frailty was added. An interesting study was performed by Kovacs et al. in the cardiosurgical population [37]. The routinely used EuroScore II had the highest predictability for postoperative death (AU ROC 0.816), followed by the Clinical Frailty Scale (AU ROC 0.778) and the Edmonton Frail Scale (AU ROC 0.738). The authors have concluded that the postoperative outcome of patients undergoing major surgery depends not only on frailty but also on the severity of any disabilities and comorbidities. In our study, the ASA classification as well as the OSS surgical risk scale and the S-MPM scale, linking surgical risk to the ASA classification, achieved acceptable results in predicting complications after general surgery. The frailty assessment scales in our data set did not perform that good.

### 4.5. In Search of the Optimal Endpoint

It is also noteworthy that the length of stay (LOS) is a frequently measured outcome considered in the context of physical decline [33,38,39,40]. Both Krishnan et al. and Cooper et al. have connected frailty with prolonged hospitalisation and discharge to institutional care after orthopaedic procedures [38,39]. A similar observation was suggested for patients undergoing lung transplantation [40]. Many confounding factors, both medical and nonmedical, should be taken into account while interpreting the above results. According to Lingsma et al., an outcome defined as the length of stay is prone to multiple aberrations on the patient level, e.g., concomitant diseases, or on the hospital level, and a composite outcome may be more accurate [41]. The discharge destination is proven to affect the length of hospital stay as well [42], primarily due to organisational obstacles. The occurrence of postoperative complications undoubtedly extends the length of hospitalisation [43,44]. It leads to overlapping of two outcomes commonly used while investigating frailty. Furthermore, Mazmudar et al. have demonstrated that LOS negatively correlates with odds for readmission after elective pancreatic resection [45], which indicates that the length of hospital stay also interferes with the readmission ratio and is not an optimal endpoint.

### 4.6. Strengths and Limitations of the Study

The advantage of this study is undoubtedly its prospective design. Researchers were able to perform frailty assessment in clinical settings, which prevented bias associated with analysis of medical records. What is more, the prospective character of our study has given the opportunity to measure frailty using CFS and EFS. Both scales require in-person evaluation of individual’s performance. In these circumstances, the application of CFS and EFS is more reliable in prospective studies, as researchers have direct insight into patients’ clinical condition. It also allows the inconveniences of deficient medical records to be avoided. Additionally, frailty assessment was carried out with three different frailty estimation models and covered a spectrum of frailty domains.

The most relevant limitation of this study is the restricted size of our cohort, which also implicates limited numbers of reported postoperative complications. Among the postoperative adverse events detected, the complications characterised as grade I to grade III in Clavien–Dindo classification predominated. The assessment of relation between frailty and perioperative mortality was precluded, as we reported only five fatal cases. In these circumstances, the results may be interfered by the insufficient sample size and the study may be underpowered. Furthermore, our study was designed to provide comprehensive assessment of frailty among individuals undergoing gastrointestinal surgeries. As a result, we observed a high level of heterogeneity of surgical procedures; the surgeries performed differed in their level of complexity and degree of surgical risk. This may potentially alter the outcomes of this research by diversifying the population included in the study. Another significant limitation is preliminary surgical assessment. We assumed that overly frail patients with remarkable surgical risk and prominent hazard of a fatal event in the perioperative period were disqualified from surgical intervention and were not included in this study. Lastly, despite special attention paid to reliable frailty assessment, the observer bias has to be mentioned. Although the Clinical Frailty Scale is considered a meaningful tool for frailty measurement, it remains the subjective evaluation of patients’ general condition and may be disturbed by researchers’ judgment.

## 5. Conclusions

ASA, S-MPM and OSS as perioperative patient classification tools perform moderately well in patients undergoing abdominal surgery. Frailty rating scales as tools for predicting perioperative complications proved suboptimal in our cohort. It is reasonable to assume that, in a more homogeneous group of initially frail patients, these tools could demonstrate greater applicability, which requires further research. It might be reasonable to develop a validated risk stratification system for elderly patients undergoing surgery, which would include criteria for organ dysfunction, complexity of surgical interventions and assessment of the patient’s frailty.

## Figures and Tables

**Table 1:** Study group characteristics.

Variable		Value
Male sex		43 (43%)
Age (years)		70 (65–74)
Height (cm)		166.5 (161.5–172)
Weight (kg)		70 (61–84.3)
BMI (kg/m^2^)		26.3 (23.6–29.7)
Obesity (BMI ≥ 30 kg/m^2^)		22 (22%)
Arterial hypertension		71 (71%)
Diabetes		18 (18%)
History of stroke or TIA		5 (5%)
Polypragmasy (≥5 medications)		40 (40%)
Type of surgery	Surgery of the pancreas	25 (25%)
	Surgery of the large intestine	25 (25%)
	Cholecystectomy	15 (15%)
	Surgery of the small intestine	9 (9%)
	Gastric surgery	8 (8%)
	Hernia repair surgery	6 (6%)
	Surgery of the esophagus	1 (1%)
	Liver surgery	1 (1%)
	Other abdominal surgery	10 (10%)
ASA-PS Class	II	34 (34%)
	III	61 (61%)
	IV	5 (5%)
Edmonton Frail Scale (EFS)	0–5 = Not Frail	67 (67%)
	6–7 = Vulnerable	19 (19%)
	8–9 = Mild Frailty	6 (6%)
	10–11 = Moderate Frailty	7 (7%)
	12 or more = Severe Frailty	1 (1%)
Modified Frailty Index (mFI)		0.18 (0.09–0.27)
Clinical Frailty Scale (CFS)		3 (2–3)

Qualitative variables are depicted as absolute value (and percentage); quantitative variables are shown as median (and interquartile range); BMI—body mass index; TIA—transient ischaemic attack, ASA-PS—The American Society of Anesthesiologists Physical Status.

**Table 2:** Postoperative medical and surgical complications by type.

Surgical Complications	Number of Patients
Anastomotic leakage	4
Eventration	2
Fluid collection at surgical site/abscess	4
Gastrointestinal bleeding	1
Gastrointestinal obstruction	1
Gastrointestinal perforation	1
Hematoma	2
Wound healing disorder	1
**Medical Complications**	**Number of Patients**
Acute Kidney injury	7
Anaemia	1
Cardiac insufficiency	7
Hemorrhagic diathesis	1
Pulmonary embolism	1
Respiratory failure	5
Sepsis and septic shock	7

Qualitative variables are depicted as absolute value; several complications may concomitantly occur in one patient.

**Table 3:** Surgical complications classification [18].

Clavien Dindo Classification of Surgical Complications Grade [18]	Number of Patients
I	5
II	11
**III**	
IIIa	2
IIIb	7
**IV**	
IVa	0
IVb	1
V	5

Several complications may concomitantly occur in one patient.

**Table 4:** Association between frailty category and in-hospital postoperative complications.

Complications/ Clavien–Dindo Classification	mFI			CFS			Edmonton		
	Non-Frail	Mildly– Severely Frail	*p*-Value	Non-Frail	Frail	*p*-Value	Non-Frail	Frail	*p*-Value
Complications (n)	2	25	0.1961	23	4	0.6159	19	8	0.6645
Medical Complications (n)	0	16	0.5572	14	2	0.5342	12	4	0.4600
Surgical Complications (n)	2	15	0.0667	15	2	0.4649	12	5	0.7311
Clavien–Dindo Classification			0.2828			0.7288			0.5727
II	0	11		9	2		8	3	
III	2	7		7	2		5	4	
IV	1	0		1	0		1	0	
V	0	5		5	0		5	0	

**Table 5:** Pearson’s correlation between the results of the scales studied.

Variables	mFI	EFI	CFS	ASA-PS	S-MPM	OSS
mFI	1.0					
EFI	0.426 *	1.0				
CFS	0.378 *	0.594 *	1.0			
ASA-PS	0.409 *	0.346 *	0.306 *	1.0		
S-MPM	0.284 *	0.356 *	0.241	0.738 *	1.0	
OSS	0.019	0.038	0.119	0.141	0.527 *	1.0

* Correlation is significant (*p* < 0.05).

**Table 6:** Frailty measures among patients with postoperative complications.

Variable	All n = 100 (100%)	Complications (In General)		*p*-Value	Medical Complications		*p*-Value	Surgical Complications		*p*-Value
		Present n = 26 (26%)	Absent n = 74 (74%)		Present n = 16 (16%)	Absent n = 84 (84%)		Present n = 17 (17%)	Absent n = 83 (83%)	
mFI	0.18 (0.09–0.27)	0.09 (0.09–0.18)	0.18 (0.18–0.21)	0.084	0.18 (0.09–0.27)	0.18 (0.18–0.18)	0.961	0.09 (0.09–0.18)	0.18 (0.18–0.18)	0.046
CFS	3 (2–3)	3 (2–3)	3 (3–3)	0.719	3 (2–3)	3 (2.6–3)	0.652	3 (2–3)	3 (3–3)	0.464
EFI	4.5 (2–6)	4 (2.25–5.75)	5 (2–6)	0.794	5 (3–5.4)	4 (3–5)	0.821	5 (2–6)	4 (3–5)	0.754

**Table 7:** Univariate logistic regression of in-hospital postoperative complications.

Demographic		Any Complications (In General)		Medical Complications		Surgical Complications	
		OR (95% CI)	*p*-Value	OR (95% CI)	*p*-Value	OR (95% CI)	*p*-Value
Age (years)		1.02 (0.96–1.09)	0.515	1.03 (0.95–1.12)	0.462	1.013 (0.934–1.09)	0.760
Sex (male)		2.48 (1.01–6.11)	0.046	0.39 (0.13–1.17)	0.093	0.34 (0.12–1.02)	0.054
BMI		1.02 (0.93–1.12)	0.698	1.04 (0.93–1.16)	0.538	1.03 (0.92–1.15)	0.640
Diabetes	Yes = 1	0.48 (0.128–1.823)	0.283	0.61 (0.12–2.94)	0.536	0.56 (0.12–2.69)	0.468
Hypertension	Yes = 1	1.23 (0.46–3.34)	0.681	1.94 (0.51–7.40)	0.331	0.70 (0.23–2.12)	0.532
COPD	Yes = 1	0.31 (0.04–2.63	0.284	0.63 (0.07–5.45)	0.677	0.59 (0.07–5.02)	0.626
ASA-PS		2.81 (1.16–6.79)	0.022	4.67 (1.45–15.16)	0.009	2.01 (0.75–5.34)	0.164
S-MPM		1.88 (1.12–3.14)	0.017	2.13 (1.08–4.19)	0.029	1.79 (1.06–3.29)	0.042
OSS		1.86 (1.05–3.31)	0.034	1.44 (0.74–2.81)	0.282	1.85 (1.04–3.63)	0.047
EFI		1.02 (0.88–1.20)	0.772	1.02 (0.84–1.23)	0.855	1.04 (0.87–1.25)	0.682
mFI		0.96 (0.92–1.01)	0.093	0.99 (0.95–1.05)	0.838	0.95 (0.90–1.00)	0.063
CFS		0.99 (0.65–1.50)	0.963	1.02 (0.62–1.68)	0.951	0.96 (0.58–1.58)	0.876

The values are ORs (with 95%CIs). CI—confidence interval. OR—odds ratio. BMI—body mass index. COPD—chronic obstructive pulmonary disease. EFS—Edmonton Frail Scale. mFI—11-factor Modified Frailty Index. CFS—Clinical Frailty Scale. ASA-PS—American Society of Anesthesiology Physical Status; S-MPM—Surgical Mortality Probability Model. OSS—Operative Stress Score.

## Data Availability

The data that support the findings of this study are available from the corresponding author (S.C.), upon reasonable request.
